# Volumetric and texture analysis of pretherapeutic ^18^F-FDG PET can predict overall survival in medullary thyroid cancer patients treated with Vandetanib

**DOI:** 10.1007/s12020-018-1749-3

**Published:** 2018-09-11

**Authors:** Rudolf A. Werner, Ralph A. Bundschuh, Takahiro Higuchi, Mehrbod S. Javadi, Steven P. Rowe, Norbert Zsótér, Matthias Kroiss, Martin Fassnacht, Andreas K. Buck, Michael C. Kreissl, Constantin Lapa

**Affiliations:** 1 Department of Nuclear Medicine, University Hospital Wuerzburg, Wuerzburg, Germany; 2 The Russell H. Morgan Department of Radiology and Radiological Science, Division of Nuclear Medicine and Molecular Imaging, Johns Hopkins University School of Medicine, Baltimore, MD USA; 3 Comprehensive Heart Failure Center, University Hospital Wuerzburg, Wuerzburg, Germany; 4 Department of Nuclear Medicine, University Medical Center Bonn, Bonn, Germany; 5 Department of Biomedical Imaging, National Cardiovascular and Cerebral Research Center, Suita, Japan; 6 Mediso Medical Imaging Systems Ltd., Budapest, Hungary; 7 Department of Internal Medicine I, Division of Endocrinology and Diabetes, University Hospital, University of Wuerzburg, Wuerzburg, Germany; 8 Comprehensive Cancer Center Mainfranken, University of Wuerzburg, Wuerzburg, Germany; 9 Würzburger Schilddrüsenzentrum, University Hospital Wuerzburg, Wuerzburg, Germany; 10 Department of Nuclear Medicine, Hospital Augsburg, Augsburg, Germany; 11 Department of Radiology and Nuclear Medicine, University Hospital Magdeburg, Magdeburg, Germany

**Keywords:** Medullary thyroid carcinoma, Tyrosine kinase inhibitor, TKI, Vandetanib, ^18^F-FDG, Positron emission tomography, 2-deoxy-2-(^18^F)fluoro-D-glucose, Personalized medicine

## Abstract

**Purpose:**

The metabolically most active lesion in 2-deoxy-2-(^18^F)fluoro-D-glucose (^18^F-FDG) PET/CT can predict progression-free survival (PFS) in patients with medullary thyroid carcinoma (MTC) starting treatment with the tyrosine kinase inhibitor (TKI) vandetanib. However, this metric failed in overall survival (OS) prediction. In the present proof of concept study, we aimed to explore the prognostic value of intratumoral textural features (TF) as well as volumetric parameters (total lesion glycolysis, TLG) derived by pre-therapeutic ^18^F-FDG PET.

**Methods:**

Eighteen patients with progressive MTC underwent baseline ^18^F-FDG PET/CT prior to and 3 months after vandetanib initiation. By manual segmentation of the tumor burden at baseline and follow-up PET, intratumoral TF and TLG were computed. The ability of TLG, imaging-based TF, and clinical parameters (including age, tumor marker doubling times, prior therapies and RET (rearranged during transfection) mutational status) for prediction of both PFS and OS were evaluated.

**Results:**

The TF Complexity and the volumetric parameter TLG obtained at baseline prior to TKI initiation successfully differentiated between low- and high-risk patients. Complexity allocated 10/18 patients to the high-risk group with an OS of 3.3 y (vs. low-risk group, OS = 5.3 y, 8/18, AUC = 0.78, *P* = 0.03). Baseline TLG designated 11/18 patients to the high-risk group (OS = 3.5 y vs. low-risk group, OS = 5 y, 7/18, AUC = 0.83, P = 0.005). The Hazard Ratio for cancer-related death was 6.1 for Complexity (TLG, 9.5). Among investigated clinical parameters, the age at initiation of TKI treatment reached significance for PFS prediction (*P* = 0.02, OS, n.s.).

**Conclusions:**

The TF Complexity and the volumetric parameter TLG are both independent parameters for OS prediction.

## Introduction

The tyrosine kinase inhibitor (TKI) vandetanib, a selective inhibitor of wild-type rearranged during transfection (RET) kinase as well as of vascular endothelial growth factor receptor (VEGFR) signaling [1–3], has demonstrated a favorable disease control in patients suffering from advanced medullary thyroid carcinoma (MTC) [4, 5]. The increased use of vandetanib outside controlled clinical settings, as well as the attendant cost and potential toxicities of the drug, underscore the need for prediction of patients who are most likely to benefit from treatment [6–8].

Volumetric assessment of tumor burden (i.e., metabolic tumor volume (MTV) or Total lesion glycolysis (TLG)) on 2-deoxy-2-(^18^F)fluoro-D-glucose (^18^F-FDG) positron emission tomography (PET) has been demonstrated as a useful tool for outcome prediction in various tumor entities, including salivary gland carcinoma, pancreatic cancer, and colorectal cancer [9–11]. As hallmarks of more aggressive disease, necrosis or hypoxia can lead to an inhomogeneous ^18^F-FDG distribution within a tumor lesion [12]. Of note, intratumoral heterogeneity derived from *in-vivo* PET reflects the heterogeneity of tracer uptake assessed by ex-vivo autoradiography [13]. Clinically, intratumoral heterogeneity (tumor texture) analysis is currently gaining ground to serve as a potential risk stratification tool in a variety of different cancer entities [14–19]. Hence, not surprisingly, combined ^18^F-FDG PET-based approaches (i.e., volumetric plus tumor texture assessment) for outcome prediction have also been investigated, e.g. in esophageal cancer [20].

In a recent study evaluating MTC patients prior to vandetanib, the prognostic potential of the metabolically most active lesion derived by baseline ^18^F-FDG PET for prediction of progression-free survival (PFS) could be demonstrated. However, this simplistic strategy failed for overall survival (OS) [21]. Hence, in the present proof of concept study, we aimed to explore, if intratumoral heterogeneity textural features (TF) as well as volumetric parameters derived from ^18^F-FDG PET prior to and 3 months after vandetanib initiation succeeded in prognostication in patients with advanced MTC.

## Material and methods

### Patient cohort

In this retrospective evaluation, a secondary analysis was performed of the identical patient cohort analyzed in [21]. Patient’s characteristics are also provided in Supplementary Table 1. All relevant clinical and outcome parameters were updated (including a novel date of censoring) and a complete re-analysis of the data was performed. All patients gave written informed consent to the diagnostic and therapeutic procedures as well as to the scientific analysis of the obtained data. Due to the retrospective nature of this analysis, the requirement for additional approval was waived by the local institutional review boards. Parts of this cohort received vandetanib in the context of a clinical trial [5].

A detailed description of the study cohort can be found in ref. [21]. In brief, between April 2007 and January 2018, 18 patients (6 females, median age at start of TKI initiation, 48 years) with advanced MTC receiving vandetanib (300 mg orally per day) were included. 14 patients were recruited at the University Hospital Würzburg, Germany, whereas the remaining 4 patients were treated at the Hospital of Augsburg, Germany. All patients had undergone various previous therapies (surgery in all patients; external beam radiation therapy in 4/18 (22.2%); chemotherapy in 3/18 (16.7%); transarterial chemoembolization in 2/18 (11.1%); sorafenib in 1/18 (5.6%) and radioiodine therapy in 1/18 (5.6%), due to an initial mis-classification as differentiated thyroid cancer). For further details refer to [21].

### Radiological Response Assessment

Response assessment was performed on a 3-months basis according to Response Evaluation Criteria in Solid Tumors (RECIST) 1.1. The best response achieved by computed tomography (CT) was also investigated (i.e., Complete Response (CR), Partial Response (PR), Stable Disease (SD) and Progressive Disease (PD)) [22]. PFS covered the time span from vandetanib initiation to the time point of RECIST-based disease progression. OS (median) was defined using the following formula: [(Date of death)–(Date of treatment initiation)] [21].

### Imaging

Imaging was performed on a stand-alone lutetium oxyorthosilicate full-ring PET scanner (ECAT Exact 47, Siemens Medical Solutions, Erlangen, Germany) in 4/18 (22.2%) of the patients (with a separate CT available in all), whereas 14/18 (77.8%) underwent integrated PET/CT (12/14, 85.7%, Biograph mCT PET/CT, Siemens Medical Solutions, Erlangen, Germany and 2/14, 14.3%, Gemini TF 16 PET/CT system, Philips Medical Systems, Hamburg, Germany). Prior to imaging, patients fasted for a minimum of 6 h (blood glucose levels < 160 mg/dl). ^18^F-FDG was administered intravenously and 60 min post-injection, transmission data were acquired from the base of the skull to the proximal thighs using 68Ge rod sources (in the case of the stand-alone PET scanner) or spiral CT. Consecutively, the PET emission data were acquired. After decay and scatter correction, the PET data were reconstructed iteratively with attenuation correction, using the algorithm supplied by the scanner manufacturer. For further details, please refer to [21]. After 3 months, another ^18^F-FDG PET/CT was performed in 16/18 (88.9%), CT in 1/18 (5.6%) and in one subject, imaging follow-up could not be obtained because of early termination of the treatment by the patient.

### Image interpretation

In contrast to the previous study, which exclusively investigated a single region of interest drawn around the metabolically most active lesion [21], a complete re-analysis of the entire tumor burden of every patient was performed: the tumor volume of all tumor lesions was determined in a consensus analysis by two board-certified nuclear medicine physicians with long-standing PET/CT experience. Volumes of Interest (VOIs) were set by using a computerized 3-dimensional volumetric rendering tool (Interview Fusion Workstation, Mediso Medical Imaging Systems Ltd., Budapest, Hungary) [23]. CT images were not used to guide delineation of the VOIs [24]. Any non-tumoral, physiological areas of ^18^F-FDG uptake were excluded. Within each VOI, the following conventional PET parameters were derived: maximum/mean standardized uptake value (SUV_max/mean_), Metabolic Tumor Volume (MTV) and Total Lesion Glycolysis (TLG). MTV was defined by volume delineation and TLG was calculated using the following formula: [MTV x SUV_mean_] [9, 25]. All conventional PET parameters as well as TF were calculated automatically by the software. The radiotracer concentration in the VOIs was decay corrected and normalized to the injected dose per kilogram of patient’s body weight to derive the SUV.

The following TF, which had been investigated in a previous study for outcome prediction in non-small cell lung cancer (NSCLC) patients under TKI treatment [19], were investigated: the first-order parameters Standard Deviation and Kurtosis, the second order parameters Entropy and Homogeneity as well as the higher-order parameters Busyness, Coarseness, Complexity, and Contrast. A detailed description can be found in [19]. In brief, first-order parameters inherit global textural features that relate to the gray level frequency distribution and are based on histogram analysis. Second-order parameters are derived from neighborhood spatial gray level dependence or co-occurrence matrices M. The matrix M determines how often a pixel with intensity i finds itself within a relationship to another pixel with intensity j in a VOI. Of note, the co-occurrence matrix describes only the changes of one voxel to the *immediate* next voxel. Higher-order parameters are calculated from 3-dimensional neighborhood gray-tone (intensity) difference matrices (NGTDM) to describe local features. These parameters are based from differences between each voxel and the neighboring voxels in adjacent image planes: The NGTDM (M4) contains *entire homogeneous areas* of a certain intensity and size [26]. Higher-order TF correlate with the human perception of tumor texture shape and therefore, reflect the human impression of an image [27].

To assess the entire baseline tumor burden, 109 metastases (median, 5 VOIs per patient, range, 2–17) were initially segmented. After treatment initiation with vandetanib, 56 metastases (median, 2 VOIs per patient, range 1–13) at follow-up were still available for segmentation. Details on segmented VOI for both baseline and follow-up PET are given in Supplementary Table 2. Change in % between both PETs was calculated using the following formula for every investigated parameter: [((Value of Follow-up PET)/(Value of Baseline PET)-1)*100].

### Tumor marker assessment and clinical parameters

Serum levels of carcinoembryonic antigen (CEA, mg/L) and calcitonin (CTN, pg/ml) were measured (prior to baseline imaging with a median of 6 determinations) [7]. Tumor marker doubling times were calculated using the American Thyroid Association Calculator [28]. Other investigated clinical parameters were metastatic sites at baseline PET, prior therapies, sex, age, and tumoral RET mutation status [21].

### Statistical analysis

Statistical analysis was performed using Medcalc (Vers. 17.4.4). The cutoff values of each parameter for PFS and OS prediction were determined by receiver operating characteristic (ROC) analysis (with the Youden-Index for maximization of specificity and sensitivity) (23). Kaplan–Meier analysis (univariate analysis) was performed using thresholds established by ROC analysis in cases in which ROC showed statistically significant results. A multivariate Cox hazard analysis was conducted to determine independent prognostic parameters (24,25). Additionally, Hazard Ratios (HR) were obtained. Statistical significance was considered with a *P* value < 0.05.

## Results

One patient had hereditary MTC. In the non-hereditary cases with available somatic RET mutational status, a somatic RET mutation was found in 3/8 cases (37.5%). ^18^F-FDG PET was positive in the entire cohort, with 17/18 (94.4%) patients presenting with lymph node involvement and 10/18 (55.6%) with lung metastases. 9/18 (50.0%) patients had liver lesions, 9/18 (50.0%) had bone lesions, 2/18 (11.1%) had soft tissue metastases, and a single subject (1/18, 5.6%) suffered from infiltration of the pancreas (Supplementary Table 1). Objective response rate was 50% with PR in 8/18 (44.4%) and CR in 1/18 (5.6%), the remainder achieved SD as best morphological response 8/18 (44.4%).

Median follow-up was 6.4 years (range, 3–10.3 y). 12/18 (66.7%) patients experienced progressive disease after a median of 2.6 y (range, 3 months–10.3 y), whereas the remaining 6 patients remained stable. Nine out of 12 progressive disease patients died of their disease (9/12, 75%) after a median of 4.1 y (range, 11 months–10.3 y).

### Imaging-based volumetric parameters and textural features derived by ^18^F-FDG baseline and follow-up PET

The mean (range) values for all investigated PET parameters are given in Table 1 (for baseline ^18^F-FDG PET, follow-up PET and change in % between both scans). Besides for Homogeneity, all investigated parameters demonstrated a decline between succeeding scans.

**Table 1:** Overview of obtained values at baseline ^18^F-FDG PET, at follow-up and change in % for the entire cohort

	Baseline	Follow-up	Change (in %)
**Parameter**	**mean (range)**	**mean (range)**	
SUV_max_	7.2 (3.4–19.9)	5.2 (2–14.11)	−27.8
SUV_mean_	3.3 (2.2–6)	2.3 (1–3.6)	−30.3
MTV (ml)	441.1 **(**64.3–3221.6)	340.2 (3.64–587.9)	−22.9
TLG	25782.6 **(**143.8–92523.9)	7427.1 (62.8–39721.2)	−71.2
Standard Deviation	1.2 (0.48–3.1)	0.8 (0.13–1.4)	−33.3
Kurtosis	0.899 (0.005–2.6)	0.891 (0.02–1.5)	−0.89
Entropy	4.1 (2.9–4.8)	3.4 (2.2–5.5)	−17.1
Homogeneity	0.4 (0.3–0.59)	0.5 (0.3–0.8)	25
Busyness	1.9 (0.24–0.6)	0.5 (0.3–0.7)	−73.7
Coarseness	0.12 (0.005–0.2)	0.1 (0.01–0.2)	−16.6
Complexity	83 (4.5–312.3)	50.5 (2.2–295)	−39.2
Contrast	13.8 (1.7–60.2)	8.5 (0.5–31.1)	−38.4

All parameters have been derived by segmentation of the entire tumor burden using volume of interests. Change in % has been calculated using the following formula: [((Value of follow-up PET) / (Value of Baseline PET) − 1) × 100].

Complexity of > 59 at baseline (in 10/18, 55.6%) correlated with significantly reduced OS of 3.3 y (vs. <59, OS = 5.3 y, 8/18 (44.4%), AUC = 0.78, *P* = 0.03; PFS, *P* = 0.07). Moreover, Contrast of >11.2 at baseline (in 9/18, 50%) was also correlated with significantly reduced PFS of 0.8 y (vs. <11.2, PFS = 4.8 y, 9/18, AUC = 0.75, *P* = 0.04; OS, *P* = 0.22).

Among the analyzed volumetric parameters, TLG > 2694 at baseline (in 11/18, 61.1%) was correlated with significantly reduced OS of 3.5 y (vs. <2694 in 7/18 (38.9%), OS = 5 y, AUC = 0.83, *P* = 0.005; PFS, *P* = 0.11).

None of the investigated delta parameters reached significance in ROC analysis. Table 2 summarizes the results of all parameters that demonstrated significance in a ROC analysis. Supplementary Table 3 gives an overview of OS and best response for every patient above/beyond the threshold for Complexity and TLG. Of note, 8/10 (80%) of the patients above the ROC-derived threshold for Complexity (for OS) were also in the high-risk as determined by the volumetric parameter TLG.

**Table 2:** Overview of results of Receiver Operating (ROC) and multivariate Cox analyses for the Textural Feature (TF) Complexity, the TF Contrast and for the volumetric parameter Total lesion glycolysis as obtained by baseline ^18^F-FDG PET

Pretherapeutic textural feature complexity								
	ROC Analysis							Cox analysis
^**18**^F-FDG PET at baseline	*p* value	Cutoff value	Sensitivity (%)	Specificity (%)	AUC	> cutoff	< cutoff	*p* value
PFS	0.07	59	66.7	83.3	0.74	1.4 y (10/18)	5.2 y (8/18)	>0.05
OS	*0.03*	59	77.8	77.8	0.78	3.3 y (10/18)	5.3 y (8/18)	*<0.0001*

Pretherapeutic textural feature contrast								
	ROC analysis							Cox analysis
^**18**^F-FDG PET at baseline	*p* value	Cutoff value	Sensitivity (%)	Specificity (%)	AUC	> cutoff	< cutoff	*p* value
PFS	*0.04*	11.2	66.7	83.3	0.75	0.8 y (9/18)	4.8 y (9/18)	*<0.04*
OS	0.22	12.9	55.6	77.8	0.67	3.8 y (8/18)	4.5 y (10/18)	>0.05

Pretherapeutic volumetric parameter total lesion glycolysis								
	ROC analysis							Cox analysis
^**18**^F-FDG PET at baseline	*p* value	Cutoff value	Sensitivity (%)	Specificity (%)	AUC	> cutoff	< cutoff	*p* value
PFS	0.11	3642	58.3	83.3	0.71	0.8 y (9/18)	4.8 y (9/18)	>0.05
OS	*0.005*	2694	88.9	77.8	0.83	3.5 y (11/18)	5 y (7/18)	*<0.0001*

Progression-Free Survival (PFS) and Overall Survival (OS) for the two groups above the cutoff (>cutoff) and below the cutoff) with the number of patients for each group are indicated

*AUC* area under the curve, *y* years

To determine independent prognosticators, a subsequent multivariate Cox analysis was performed: for PFS, baseline TF Contrast was found to be significant (*p* = 0.04). For OS, baseline TF Complexity and baseline TLG reached significance (*p* < 0.0001, respectively).

For those subjects above the ROC-derived threshold, the HR for Complexity was 6.1 for OS (CI, 1.6–23.7; PFS, 2.9, CI, 0.9–9.2). Similar results could be obtained for the TF Contrast for patients above the threshold: HR was 3.7 (1.1–12.2) in terms of PFS (OS, 1.9, CI, 0.5–7). For the volumetric parameter TLG, the HR was 9.5 (CI, 2.6–35.2) for OS (PFS, 2.3, CI, 0.7–7.3). Among investigated clinical parameters, the age at initiation of TKI treatment demonstrated significance for PFS prediction (*p* = 0.02; OS, n.s.).

**Fig. 1:**
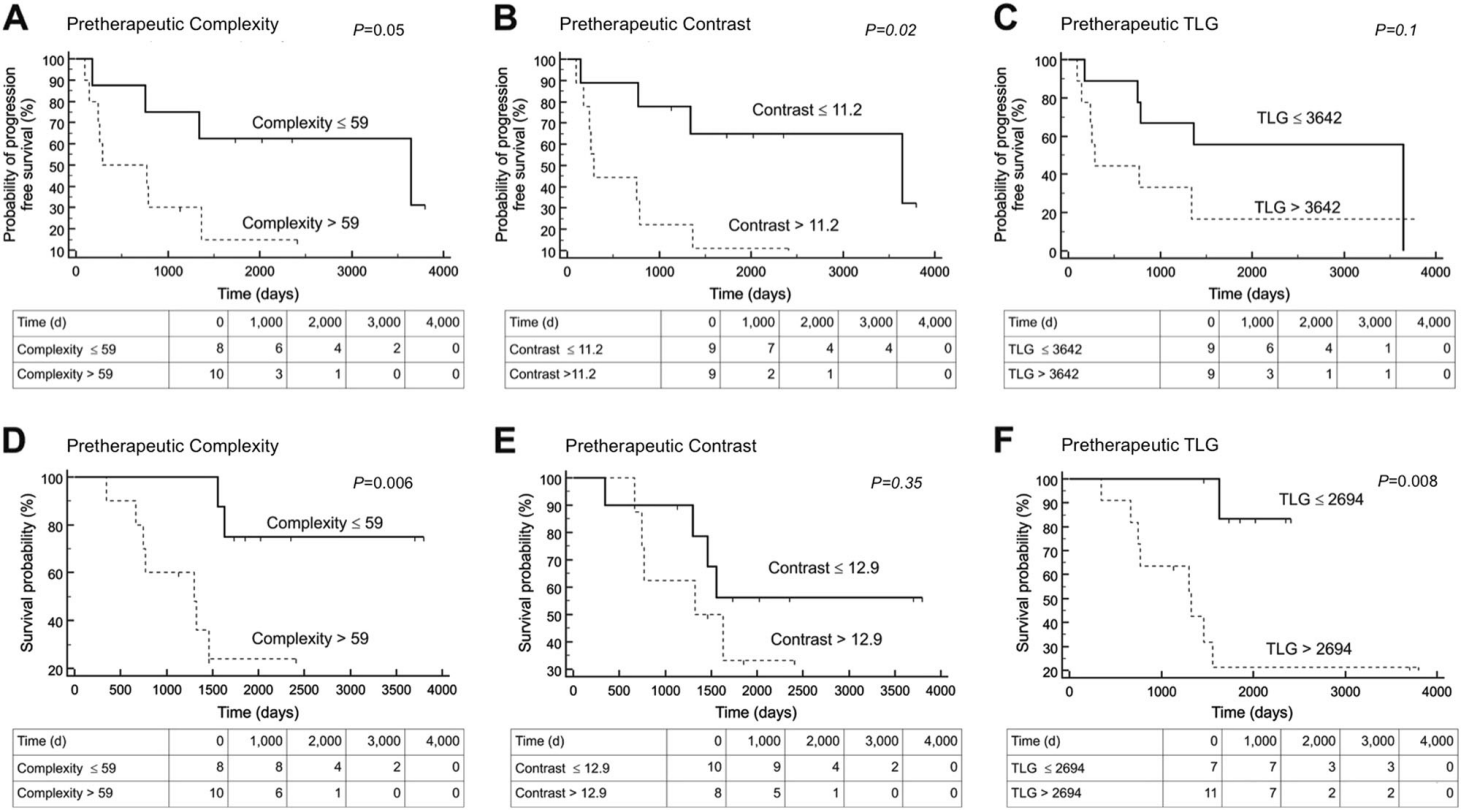
Kaplan–Meier plots for the probability of progression-free (PFS, upper row) and overall survival (OS, lower row) for the Textural Feature (TF) Complexity **a**, **d**, as well as for the TF Contrast **b**, **e** and for Total lesion glycolysis (TLG, **c**, **f**) Low-risk group (solid lines) and high-risk groups (dashed lines) could be identified by analysis of pretherapeutic ^18^F-FDG PET prior to TKI initiation. *P* values of Kaplan–Meier analyses are displayed for each parameter. Cutoff values obtained by Receiver Operating Characteristics Analysis (Table 2) were used. Pretherapeutic Contrast reached significance for PFS in both ROC and Cox analysis, while pretherapeutic Complexity and TLG were significant for OS in both statistical tests

Using ROC-derived cutoffs, Kaplan–Meier analysis found a significant separation between high- and low-risk groups for baseline TF Contrast (PFS, *p* = 0.02). For OS, baseline TF Complexity (*p* = 0.006) and baseline TLG (*p* = 0.008) reached significance. Respective Kaplan–Meier Plots for selected parameters (all obtained from baseline ^18^F-FDG PET) are displayed in Fig. 1.

## Discussion

In our previous investigation, SUV_mean_ of a pretherapeutic ^18^F-FDG PET derived by a simplistic region of interest was able to predict PFS for MTC patients undergoing treatment with vandetanib. However, this conventional approach of solely focusing on the metabolically most active lesion failed to reliably predict OS [21]. Hence, in the present study, the entire tumor burden derived by baseline and follow-up PET was re-analyzed. Further, the prognostic potential of tumor heterogeneity was evaluated. Specifically, the higher-order TF Complexity was found to be an independent predictor of patient outcome. Complexity, derived by the neighborhood gray-tone difference matrices, mainly represents a high degree of information content (i.e. an increased number of patches and primitives obtainable in the analyzed texture) [19]. Not surprisingly, Complexity derived by baseline PET portended inferior outcome with significantly reduced OS and trended towards significance for PFS in a ROC analysis (*P* = 0.07). These findings were further corroborated by obtaining an HR > 6 for cancer-related death for those subjects above the ROC-derived threshold. Similar results were observed for the volumetric parameter TLG: a higher glucose consumption by the tumor volume at baseline was also independently associated with shortened OS (HR, 9.5; Table 2).

Changes in PET features for TKI response assessment and survival prediction have been evaluated in numerous previous studies. However, primarily SUV parameters (SUV_peak_, SUV_max_) have been investigated for outcome prediction, e.g., in NSCLC treated with the TKI erlotinib [29–31]. Cook et al. reported on heterogeneity assessment in NSCLC treated with the same TKI and the change of first-order parameters were correlated with survival [19]. However, due to a higher extent of voxel intensities, parameters of first-order intrinsically have a higher risk of association with SUV, particularly in patients with increased SUV_max_ [19, 26]. Of note, the range of SUVs derived by the entire tumor burden was rather low in the present investigation (SUV_max_ at baseline, 3.4–19.9 vs. SUV_max_ at baseline in the study of Cook et al., 1.2–30.1 [19]). Consequently, no association between first-order TF and outcome was found in our study. An impact of tumor size on NGTDM-derived TF (such as Complexity) cannot be definitively ruled out [32]: an increase in tumor volume might be associated with an increase in necrosis/hypoxia, which ultimately results in higher informative content within a VOI. However, Complexity was significant in both ROC and Cox analysis for OS, which emphasizes its independent statistical value from other tumor-size related parameters, such as MTV. Notably, discordances between (morphological) responses and outcomes as assessed by textural features could be recorded. Only baseline parameters yielded prognostic value. Given the small number of included patients, the different scanners and the variety of the imaging protocols that have been used in the present study, no firm conclusions can be drawn yet. Taken together, further research with a larger number of advanced MTC patients, preferably on one scanner, is definitely warranted.

RET mutations in MTC demonstrate distinctive mutational heterogeneity and the mutational status can differ between primary tumor and other lesions and even among synchronous or metachronous metastases [33]. Although in the present study, a RET mutation could only be proven in a minority of the cases (3/8, 37.5%), these different oncogenic mechanisms underscore the complexity of tumor biology in progressive MTC. Additionally, vandetanib *per-se* leads to inhibition of tyrosine kinases resulting in reduced tumor proliferation and angiogenesis [34] and novel approaches to further investigate this potential dedifferentiation under treatment are being intensively sought. Several studies have hypothesized that intratumoral heterogeneity derived by PET or CT is linked to angiogenesis or hypoxia [35]: both factors are known to be histopathological correlates denoting either true tumor escape or more aggressive tumor behavior [36]. Although the herein presented strategy of prognostication by using PET-based tumor texture in advanced MTC should be rather interpreted as a “proof-of-concept”, it might open avenues to identify high-risk patients at an earlier stage or to monitor those patients more closely.

The herein obtained data must be interpreted with extreme caution: First, although a homogenous cohort has been studied, the number of included patients in the present investigation is rather low, thus limiting statistical power. A prospective trial on a larger scale could further strengthen our preliminary findings. Second, different scanners at different treatment sites were used and therefore, imaging procedures varied from center to center. Thus, an impact on semi-quantification and on the reproducibility of the derived findings cannot be excluded. Apart from that, manual segmentation itself is prone to observer bias; however, experienced nuclear medicine physicians have determined the tumor volume in a consensus analysis. Moreover, the herein presented parameters should be easily obtainable, e.g. by a (semi-)automatic lesion detection software: this might pave the way for a cost-effective and time-saving implementation of TF assessment in clinical routine in the long run [37]. Future efforts may also investigate whether and to what extent pre-treatment (e.g. by loco-regional procedures) has an impact on intra-tumoral heterogeneity and outcome correlations.

## Conclusions

The TF Complexity and the volumetric parameter TLG, obtained from baseline PET, are both independent parameters for OS prediction in MTC patients scheduled for TKI treatment. Further investigations using intra-tumoral heterogeneity for risk stratification and prognostication are warranted, particularly in patients suffering from tumor entities treated with TKI.

## Electronic supplementary material

Supplementary Material
