# Supplementary material for: Volumetric and texture analysis of pretherapeutic 18F-FDG PET can predict overall survival in medullary thyroid cancer patients treated with Vandetanib
Source: Endocrine. 2018 Sep 11;63(2):293–300. doi: 10.1007/s12020-018-1749-3 (PMC6394453; doi:10.1007/s12020-018-1749-3)
Supplement: Supplementary file 1 — Supplementary Material [file 12020_2018_1749_MOESM1_ESM.docx]

**SUPPLEMENTARY TABLES**

**Volumetric and Texture Analysis of Pretherapeutic ^18^F-FDG PET can Predict Overall Survival in Medullary Thyroid Cancer Patients Treated with Vandetanib**

Rudolf A. Werner^1,2,3,*^, Ralph A. Bundschuh^4,*^, Takahiro Higuchi^1,3,5^, Mehrbod S. Javadi^2^, Steven P. Rowe^2^, Norbert Zsótér^6^, Matthias Kroiss^7,8,9^, Martin Fassnacht^7,8,9^, Andreas K. Buck^1,3^, Michael C. Kreissl^10,11,#^, Constantin Lapa^1,#^

1. Department of Nuclear Medicine, University Hospital Wuerzburg, Wuerzburg, Germany;

2. Johns Hopkins University School of Medicine, The Russell H. Morgan Department of Radiology and Radiological Science, Division of Nuclear Medicine and Molecular Imaging, Baltimore, MD, United States;

3. Comprehensive Heart Failure Center, University Hospital Wuerzburg, Wuerzburg, Germany;

4. Department of Nuclear Medicine, University Medical Center Bonn, Bonn, Germany;

5. Department of Biomedical Imaging, National Cardiovascular and Cerebral Research Center, Suita, Japan;

6. Mediso Medical Imaging Systems Ltd., Budapest, Hungary;

7. Department of Internal Medicine I, Division of Endocrinology and Diabetes, University Hospital, University of Wuerzburg, Wuerzburg, Germany;

8. Comprehensive Cancer Center Mainfranken, University of Wuerzburg, Wuerzburg, Germany;

9. Würzburger Schilddrüsenzentrum, University Hospital Wuerzburg, Wuerzburg, Germany;

10. Department of Nuclear Medicine, Hospital Augsburg, Augsburg, Germany;

11. Department of Radiology and Nuclear Medicine, University Hospital Magdeburg,

Magdeburg, Germany.

^*,#^ = contributed equally to this work.

*Running head:* Texture Analysis of ^18^F-FDG in MTC

*Word count:* 2,945

*Corresponding author:*

Rudolf Werner, MD, Johns Hopkins University School of Medicine, The Russell H Morgan Department of Radiology and Radiological Science, 601 N Caroline Str., JHOC 3230, 21287 Baltimore, MD, U.S.

Phone: +1 443 430 4905, E-mail: [rwerner3@jhmi.edu](mailto:rwerner3@jhmi.edu)

| Case | Sex | Age (y) | Metastatic sites | Disease type | Prior therapy | Somatic RET mutation |
| --- | --- | --- | --- | --- | --- | --- |
| #1 | f | 57 | LN, lung, liver | sporadic | surgery | unknown |
| #2 | f | 59 | LN, liver | sporadic | surgery, CTx,  TACE | negative |
| #3 | m | 41 | LN, bone | sporadic | surgery, CTx | unknown |
| #4 | m | 50 | LN, lung | sporadic | surgery | unknown |
| #5 | f | 20 | LN, lung, liver | sporadic | surgery | negative |
| #6 | m | 57 | LN, lung, liver, bone | hereditary | surgery, TACE | - |
| #7 | m | 40 | LN, lung | sporadic | surgery, CTx | unknown |
| #8 | m | 40 | LN, liver, bone | sporadic | surgery | negative |
| #9 | f | 35 | LN, lung | sporadic | surgery | negative |
| #10 | m | 59 | LN, lung | sporadic | surgery | unknown |
| #11 | m | 30 | LN | sporadic | surgery, RTx | negative |
| #12 | m | 47 | LN, liver, bone, soft tissue, pancreatic infiltration | sporadic | surgery | unknown |
| #13 | m | 54 | LN, lung, liver | sporadic | surgery | unknown |
| #14 | m | 78 | LN, lung, bone | sporadic | surgery | unknown |
| #15 | f | 49 | LN, liver, bone | sporadic | surgery, RTx | positive |
| #16 | f | 28 | LN, bone | sporadic | surgery, radioiodine therapy*, RTx | positive |
| #17 | m | 46 | liver, bone | sporadic | surgery, sorafenib | unknown |
| #18 | m | 55 | LN, lung, bone, soft tissue | sporadic | surgery, RTx | positive |

**Supplementary Table 1.** Detailed patients` characteristics. CTx = chemotherapy, f = female, LN = lymph node, m = male, RET = rearranged during transfection, RTx = radiation therapy, TACE = transarterial chemoembolization, y = years. * initially classified as differentiated thyroid carcinoma. Modified from Werner et al. [21] © by the Society of Nuclear Medicine and Molecular Imaging, Inc.

| VOI set on | BASELINE ^18^F-FDG PET  (number of VOI, (in %))  n(total)=109 (median, 5 p. P.) | FOLLOW-UP ^18^F-FDG PET  (number of VOI, (in %))  n(total)=56 (median, 2 p. P.) |
| --- | --- | --- |
| Lymph Nodes | 47/109 (43.1) | 29/56 (51.8) |
| Bone Lesions | 39/109 (35.8) | 18/56 (32.1) |
| Lung Lesions | 11/109 (10.1) | 2/56 (3.6) |
| Soft tissue Lesions | 7/109 (6.4) | 4/56 (7.1) |
| Liver Lesions | 5/109 (4.6) | 3/56 (5.4) |

**Suppplementary Table 2.** Overview of Volumes of Interests (VOI) placed on tumor lesions on a baseline and a follow-up ^18^F-FDG PET to derive the entire tumor burden prior to and after vandetanib initiation (by using a 3-D volumetric rendering tool). p. P. = per patient.

|  | | BASELINE PET FOLLOW-UP PET | | | | | CHANGE (in %) | | | RESPONSE/SURVIVAL | | | |  |
| --- | --- | --- | --- | --- | --- | --- | --- | --- | --- | --- | --- | --- | --- | --- |
| Case | | **Complexity** | **TLG** | **Complexity** | | **TLG** | **Complexity** | | **TLG** | | **Best Response^#^** | **PFS** | **OS** | |
| #1 | 16.2 | | 3642.3* | 7.9 | 62.8 | | -51.2 | -98.3 | | | PR | n/r | s/a | |
| #2 | 194.1* | | 30025.9* | 48.5 | 25832.4 | | -75 | -14 | | | SD | 8 | 25 | |
| #3 | 312.3* | | 6788.5* | 14.9 | 2941.7 | | -95.2 | -56.7 | | | SD | 8 | 21 | |
| #4 | 28.6 | | 2694.01* | 5.4 | 336.2 | | -81.1 | -87.5 | | | PR | 119 | s/a | |
| #5 | 59.1* | | 627.21 | 106.5 | 168.9 | | 80.2 | -73.1 | | | PR | n/r | s/a | |
| #6 | 25.9 | | 143.83 | 6.4 | 160.5 | | -75.3 | 11.6 | | | SD | 5 | 53 | |
| #7 | 100.2* | | 3030.9* | 17.6 | 140.6 | | -82.4 | -95.4 | | | SD | 44 | 48 | |
| #8 | 60.1* | | 92523.9* | 67.2 | 36085.8 | | 11.8 | -61 | | | PR | 25 | 42 | |
| #9 | 7.6 | | 227.2 | 10 | 114.7 | | 31.6 | -49.5 | | | PR | n/r | s/a | |
| #10 | 4.5 | | 6170.5* | 46.5 | 307.7 | | 933.3 | -95 | | | SD | 44 | 51 | |
| #11 | 35.3 | | 805.7 | 295.1 | 420.6 | | 735.9 | -47.8 | | | PR | n/r | s/a | |
| #12 | 86.1* | | 278661.8* | 6.5 | 39721.2 | | -92.4 | -85.7 | | | PR | 4 | 11 | |
| #13 | 22.4 | | 449.5 | n/a | n/a | | n/a | n/a | | | SD | 24 | s/a | |
| #14 | 196.7* | | 4352.1* | n/a | n/a | | n/a | n/a | | | n/a (side effects) | 3 | 24 | |
| #15 | 131.3* | | 20885.2* | 40.1 | 1592.9 | | -69.5 | -92.4 | | | PR | 9 | 43 | |
| #16 | 112.9* | | 8722.8* | 128.9 | 9779.3 | | 14.2 | 12.1 | | | SD | n/r | s/a | |
| #17 | 15.5 | | 2532.3 | 2.2 | 752.4 | | -85.8 | -70.3 | | | SD | n/r | s/a | |
| #18 | 85.1* | | 1802.8 | 4.9 | 415.7 | | -94.2 | -76.9 | | | CR | 26 | s/a | |

**Supplementary Table 3. Values for the Textural Feature Complexity, the volumetric parameter Total Lesion Glycloyis (TLG), the changes in % as well as Response Evaluation (Best Response), Progression-Free (PFS) and Overall Survival (OS) given for every case in months (in accordance to the updated date of censoring).** Change in % has been calculated by using the following formula: [(Value of Follow-up PET)/(Value of Baseline PET)-1)*100]. CR = Complete Response, PET = positron emission tomography, PD = Progressive Disease, PR = Partial Response, SD = Stable Disease. n/a = not available; n/r = not reached at date of censoring; s/a = still alive at date of censoring. * = above the cut-off value which had reached significance according to receiver operating characteristic analysis for OS (see Table 2: baseline Complexity, 59 and baseline TLG, 2694). ^#^ = best response achieved by computed tomography criteria (Response Evaluation Criteria In Solid Tumors 1.1) during follow-up [21].
